# Hospital and Institutional News

**Published:** 1912-08-03

**Authors:** 


### HOSPITAL AND INSTITUTIONAL NEWS.

### THE DOCTORS' VOTE IN MANCHESTER.

The North-West Manchester by-election is interesting to the hospital world from the definite action which the medical profession is taking. On Tuesday night the North Manchester division of the British Medical Association issued a manifesto appealing "to those who have received assistance from the medical profession in the past . . . to use every lawful means in their power to secure the overwhelming defeat of the Government nominee in the election which is now pending, and thus indicate their disapproval of the unseemly haste and consequent crudities of the National Insurance Act." The solidarity of the profession has been so remarkable hitherto that this manifesto may effect the medical vote *en bloc*. Exactly what strength this may prove to be at the polls it is not easy to calculate, but that even a numerically small section might prove of some importance is clear from the fact that the Government's majority at the last election was 445.

### THE KING'S FUND AND OUT-PATIENTS.

The Report of the Committee which was appointed in January 1911 by the Governors of King Edward's Hospital Fund for London to inquire into "the system prevailing in the London hospitals with regard to the admission of out-patients" was issued last week. The Committee, which consisted of Lord Mersey, the Bishop of Stepney, and Lord Northcote, who died last year, thus concludes with only two signatures. Twenty-seven meetings were held, and the witnesses represented twenty-one hospitals, while written statements were received from forty-four others. In their general conclusions great emphasis is laid on the work of the hospital almoner, as the following summarised quotation will show: To organise an out-patient department which will distinguish between the different classes of applicant and co-operate with other medical and charitable agencies, both in referring patients to them and in accepting patients from them, demands the employment of inquiry officers capable of something more than the mere prevention of abuse. Such officers already exist in the hospital almoners. Such an organisation of the out-patient department, the Committee think, falls in with a just conception of the proper sphere of hospital assistance. Therefore, the Committee recommend the extension of the almoner system to cover all the out-patient departments. It helps to exclude from the out-patient departments the class able to pay for medical attendance, the provident dispensary class, and the Poor-Law class. It supplements hospital assistance where necessary, and it encourages the reference to the hospital by other agencies (including private practitioners) of cases needing specialist advice or treatment.

### THE ALMONER AS THE PIVOT OF REFORM.

One main object, the Report proceeds, of out-patient organisation should be to promote co-operation between the hospital and general practitioners, and the general adoption through almoners of co-operation with general practitioners would go far to remove the grievances which at present so often deter the private doctor from sending cases to hospitals for a second opinion or for special treatment. It is most important that a general uniformity in the principles and methods of out-patient administration should be adopted in all the hospitals of London. Without this there would be either injustice as between one hospital and another and between one locality and another, or else the whole system would be likely to relapse into the former condition of disorganisation. It is evident that a thorough system of out-patient organisation would involve a temporary increase in total expenditure and a permanent increase in the cost per patient. But the same arguments which justify expenditure upon improved methods of medical treatment may be used to justify expenditure upon improved methods of charitable administration. The Committee say that they have not considered the possible effect of the Insurance Act on the out-patient departments of the London hospitals.

### AN ARCHBISHOP ON MARRIAGE AND DISEASE.

Annulment of marriage is not often preached by the clergy, who are seldom anything but deaf to the scientific reasons for which on occasion it may be urged, and therefore it is gratifying to be able to publish the Archbishop of York's weighty pronouncement on the subject in his inaugural address as President of the Royal Sanitary Institute at York last Monday. "I declare myself," his Grace remarked, "in favour of an amendment of the marriage laws providing that marriages should be declared null and void if, within a certain time, it is shown that facts have been withheld discovering insanity, epilepsy, or venereal disease." In spite of their caution these remarks are noteworthy and will earn the applause and gratitude of medical men. In them the profession will recognise an awakening on the part of Churchmen to the lessons which medicine and science have established, and to the facts which great investigators like Havelock Ellis have put on record and talented men like Brieux have popularised. Once get the principle admitted that the presence of specific diseases may be a factor sufficient to annul marriage and the first step has been taken towards remedying the worst abuses of the present marriage laws, abuses which hitherto the medical profession alone has fully realised. The co-operation of the Archbishop of York should do much to awaken the public conscience to the facts. The York meeting of the Royal Sanitary Institute has certainly opened well.

### PHARMACISTS IN CONFERENCE.

Much of the progress which pharmacy has made during the past half-century can be credited to past and present members of the British Pharmaceutical Conference, an organisation which was established nearly fifty years ago for the purpose of encouraging the study of pharmacy in its higher branches.

It is of interest to mention that hospital pharmacists have always played a prominent part in connection with the work of the Conference. Of the two secretaries who arranged the scientific programme for this week's meeting one, Mr. H. Finnemore, B.Sc., F.I.C., is chief pharmacist to Guy's Hospital, while both secretaries for next year will be hospital pharmacists, since Mr. Finnemore will have for his colleague Mr. R. R. Bennett, B.Sc., F.I.C., chief pharmacist to University College Hospital. The President, Sir Edward Evans, in his opening address dealt with the question of the cultivation of drugs, and he suggested that the Government might with advantage form in the Board of Agriculture a department such as exists in the United States to further the growth in this country of herbs and roots. The facts show the soil of this country to be adaptable for this purpose, since those it does produce—digitalis, henbane, colchicum, valerian, belladonna, peppermint, lavender, etc.—are superior to any produced elsewhere. The same subject was dealt with in greater detail by Mr. J. H. E. Evans. In a paper on "The Formaldehyde Solution and Tablets of Commerce" it was shown by Mr. C. H. Hampshire, B.Sc., and Mr. S. Furnival that many of the latter on the market contain much less formaldehyde than is required by the formula of the British Pharmaceutical Codex. The preparation of bacterial vaccines was described in a paper by Dr. Ian Struthers Stewart, who said that in the treatment of diseases of bacterial origin vaccines promised to oust all other remedies. In a communication from the Pharmaceutical Laboratory of Guy's Hospital Mr. H. Finnemore and Miss Dorothy Braithwaite stated that some time ago the observation was made that when ether is added to a concentrated extract of ipecacuanha a crystalline precipitate is produced. This is a glucoside, the pharmacological effect of which has been subjected to a preliminary study by Dr. G. W. Goodhart. Mr. R. R. Bennett put on record some experiments undertaken to determine the solubility in normal saline of pure ethyl oxide and the solubility of the purified "methylated" ether which is in general use for anæsthetic purposes; as the intravenous administration of ether dissolved in normal saline solution has recently engaged the attention of anæsthetists, Mr. Bennett's paper is a useful one.

### FIRE AT AN ISOLATION HOSPITAL.

What might have proved a disastrous fire broke out in the dispensary at Thirsk Isolation Hospital, Yorkshire, last week. A paraffin lamp was upset, the oil, itself alight, set fire to several articles, and at one time seemed to have caught hold of the match-boarding which lines the interior of the building. The patients, many of whom have come from Norby, where there has been a typhoid epidemic, were removed from the adjoining part of the hospital, and the fire was successfully extinguished by the staff. The hospital is a corrugated-iron building, the light construction of which renders accidents of this kind of some danger, though, in the net result, the amount of damage actually suffered was fortunately small.

### THE LONDON HOSPITAL AND TREATMENT CENTRES.

The Education Committee of the London County Council have again been in communication with the Board of Education as to their medical treatment schemes. The Board state that they are prepared to regard as satisfactory arrangements of the kind in operation at the London Hospital, where there is within the hospital what is practically a treatment centre for school children. The arrangements in force at certain other hospitals, which differ not only in degree but in kind from the arrangements made at the London Hospital, could not be regarded as satisfactory. If the arrangements at these other hospitals could be brought into line with the arrangements at the London Hospital the Board would be prepared to regard them as a satisfactory part of the Council's scheme of treatment. Failing this, they look forward to a gradual transfer of the work either to hospitals where the arrangements are of the London Hospital type, to medical treatment centres, or to school clinics. The Education Committee are considering the question of establishing in connection with the hospitals with which the Council has agreements a system which will produce the same results as obtain at the London Hospital.

### CHINESE PATIENTS AND WESTERN SURGERY.

An interesting Colonial Office report on Weihaiwei informs us that a new hospital was established at Wênch'uant'ang, where the district officer resides, in buildings which belong to the neighbouring villages. It was intended to place in charge of it the Chinese medical assistant, who had been under the medical officer on the island, as it was thought that his services could be much more usefully employed among the Chinese in the territory. He, however, resigned, but the Government was fortunate in securing, through Dr. Neal, of the American Presbyterian Mission Hospital at Tsinanfu, the capital of the province of Shantung, the services of Mr. Ma, who has had twenty years' experience of medical work in that hospital under Dr. Neal. In order to encourage the Chinese to make use of this hospital and the services of the medical assistant in charge of it, no charge of any kind was made for medical attendance, treatment, or drugs. The result has been most satisfactory, for during a period of less than eight months 4,844 patients applied for medical aid, the number of attendances being 9,054. This remarkable success is in a large measure due to the personality of Mr. Ma, who by his courtesy and kindly manner has gained the confidence and esteem of the Chinese. Mr. Ma has been especially successful in dealing with cases of attempted suicide. As the Chinese have shown themselves only too ready to make use of the new hospital, it has been decided in future to make a charge for drugs supplied to patients who can afford to pay for them, an arrangement which is approved by the Chinese, for the benefit of whom the hospital has been established. The number of operations was fifty-nine. Amongst them were three successful cases of abdominal section, the satisfactory result of which caused great surprise among the Chinese interested, and moved some of them to shew their appreciation and gratitude by presenting to Dr. Muat, medical officer, a tablet to be hung in the Port Edward Hospital, on which are inscribed Chinese characters meaning that through his skill those who were about to die have been restored to life.

### WOMEN IN PHARMACY.

The appointment of women to posts previously held by men is becoming a familiar feature of our age. In no profession, perhaps, is this more noticeable than in dispensing, especially in that branch of the work which concerns public institutions. So long as the standard for efficient qualification is kept high and there is no unfair reduction of salary there is small cause for complaint. We note this week that Miss F. Bertha Prince has been appointed pharmacist to the Nottingham General Hospital, a post formerly held by the late Mr. W. H. Crackle. Miss Prince, who qualified as a chemist and druggist in 1908, had been engaged as assistant dispenser for some years in the service of the same committee which has now entrusted her with the more responsible position.

### THE ROYAL FREE HOSPITAL'S NEW DEPARTMENT.

Considerable progress in the preliminary arrangements for a new out-patients' department at the Royal Free Hospital is now being made. Upwards of an acre of land has been acquired immediately adjoining the rear of the hospital, and sketch-plans prepared by Mr. H. V. Ashley have been approved. Possession of the land is to be given on September 29, after which date no delay will be incurred in the preparation of working drawings and the details of the accommodation to be provided. The scheme proposed to be carried out will be considered by the Committee of King Edward's Hospital Fund, and, as very great care and thought have been bestowed upon the matter before submission, the authorities hope it is almost impossible that any improvements could be suggested. The accommodation to be provided will include on the ground floor a casualty block, gynæcological, physicians', surgeon, throat, and eye departments, a dispensary, and the almoner's office. On the first floor the dental, massage, electrical, and *x*-ray departments will be situated, students' and wardmaids' quarters, and a suite of rooms for the nursing staff connecting by means of a private staircase with similar nurses' quarters on the floor above. On this floor provision is made for a complete maternity department, comprising one ward of eight beds, labour ward of three beds, theatre, and offices. Connected with this department is a residential portion allotted to the obstetric staff. It is, of course, only after protracted negotiations that the land has been secured on which to build, and in the meantime the necessity for a new out-patients' department has been rendered more urgent. Although a large expenditure will be involved, the authorities hope that the cost of annual maintenance will not be increased unduly by reason of the additional buildings, as several economies are possible by concentrating the departments now provided without as well as within the area of the present hospital buildings.

### LONDON'S MEDICAL OFFICER AND THE INSURANCE COMMITTEE.

The London County Council on July 30 declined a request from the Insurance Committee for the County of London that the Medical Officer of Health for the County, Dr. Hamer, should attend meetings of the Committee. The grounds of this refusal were that all the time and energies of the medical officer were necessarily devoted to the work of his office, and that the Council had already appointed as a member of the Insurance Committee Sir Shirley Murphy, the late county medical officer, whose consultative services had been retained. The Committee felt confident that his great knowledge and experience would supply all that the Committee required. No other county or borough council has refused to allow its medical officer to assist the local Committee.

### THE LORD MAYOR'S ADVICE TO HIS PROFESSION.

In visiting the West London Hospital last week to be elected an honorary member of the Medico-Chirurgical Society, Sir Thomas Boor Crosby, M.D., Lord Mayor of London, urged the medical profession to take fuller part in public life, and drew the moral from his own activities. He said that the medical profession had too long held aloof from corporate and parochial duties, to the detriment of the truest and best interests of their profession. He hoped that this apathy would not continue, and that they would not fail in future to assist in those public duties for which, from their educational and analytical ideas, they were peculiarly well fitted. Mr. McAdam Eccles, who presided, said how much the dignity of the profession had been enhanced by one of its members acting as Chief Magistrate of the City.

### THE FEES OF HOSPITAL ARCHITECTS.

In a report of his audit of the Haddon District Hospital Committee's accounts, Mr. W. N. Hunt, the Local Government Board inspector, criticises the amount which the committee have spent on architects' fees. After noting that during the year Messrs. Bryden and Walton, architects, submitted an account for £154, being their charges for preparing plans, etc., in connection with the erection of the proposed isolation hospital at Edgestone Lane, near Ashford, for a period extending from October 1907 to July 1909, and that they eventually consented to accept £118 in full discharge, he comments as follows: "It is, I consider, very regrettable that since the committee were first constituted such a considerable amount has been expended in architects' fees, etc., without any satisfactory result having been obtained." The inspector's strictures, however, must be read in the light of the local statement that twelve years have been spent by the committee in considering the erection of a hospital. What might appear at first sight to be a criticism of architects is thus shown to be a criticism of a hospital committee, which should be compelled to show cause why this time and this expense has been incurred without any satisfactory result having been obtained. A building committee's indecision is the opportunity of the architect.

### THE LEEDS INFIRMARY EXTENSION SCHEME.

A curious use is being made of an inference drawn from the incidence of the Insurance Act at Leeds, where, as was probably inevitable, a certain number of persons are criticising the City Council's proposal to spend £131,000 on certain public improvements, which we have outlined already, in connection with the Leeds Infirmary Extension Scheme. It is urged, and may be repeated at a forthcoming meeting of the Council, which is expected to be held this month, that the Insurance Act will cause so many voluntary subscriptions to be withdrawn that the municipality will have to provide £15,000 or £20,000 a year in order to maintain the infirmary in its extended form. This argument is so transparently a piece of special pleading, so obviously insincere, that it need not be seriously considered, but that it should be made at this time and for this purpose is instructive as showing to what lengths obstructionists will go in attempts to support a bad case. An amending Act is very desirable, and even now may very possibly prove necessary, but the solid opinion which the Extension Scheme has been able to attract will see in this latest argument only a tribute to the strength of its position.

### A MATRON AND DISPENSER PRAISED FOR ECONOMY.

Satisfactory financial progress was revealed at the half-yearly meeting of the Essex County Hospital. Mr. H. B. Dickinson, indeed, was able to announce that rare combination—an increase of income and a decrease of expenditure. Annual subscriptions have improved, and, in spite of an increase in the number of patients, the provision bill is £60 smaller than it was last year. "Surgery and dispensary charges" have fallen from £496 to £345. Dr. S. W. Curl, in commenting on this reduction, remarked that a large share of credit belonged to the hospital's dispenser, as the saving indicated previous unnecessary expenditure. He added also that the matron deserved credit for the saving affected under "domestic" charges. To this, however, the matron modestly replied that the economy was largely due to the improved results of the "Pound Day" collection. No better instance could be found of the value of "Pound Day." The economies which it effects are the true measure of its usefulness.

### THE RETIREMENT OF MR. CHARTER SYMONDS.

Mr. Charter Symonds, to universal regret, is retiring from the post of senior surgeon to Guy's Hospital. Of Mr. Symonds' great reputation both as a teacher and technician there is no need to speak, but it is well to place on record that these qualities are enhanced by another which caused him to be described more than once as "the surgeon who does not operate." Mr. Symonds never allowed the patient's true interests to be overlooked, and never advised operations unless he had satisfied himself that the chance of ultimate recovery or benefit well outweighed the risk involved. If these qualities are valuable in the practising surgeon, and they deservedly placed him in his high position as *facile princeps* the patient's practitioner, they are doubly so in a teacher who occupies a position which is bound to influence the younger men. That is why this retirement marks an important date, and why regret is so sincere, and why so many gathered, besides the students, to honour him at his last class on Monday.

### A BOARD OF CONTROL FOR THE FEEBLE-MINDED.

An important Government amendment to the "Mental Deficiency Bill" was carried, on Mr. McKenna's motion, at the meeting of Standing Committee B of the House of Commons last Monday. Mr. McKenna moved to substitute a Board of Control as a central authority instead of the Secretary of State's Department. In supporting this proposal, he reminded the Committee that in the Bill as carried on second reading the Government proposed to set up a second body of Commissioners in addition to the Lunacy Commissioners, the function of the new body being to deal exclusively with feeble-minded persons. The objection was raised that it was an unbusiness-like proceeding to set up a new body and not take advantage of the experience which the Lunacy Commissioners had already acquired. What was now proposed was to establish a single Board of Control. The Board would consist of not more than fifteen members, of whom twelve would be paid. There were now eight paid Commissioners, so that the net increase of paid members would be four, at least one of whom would be a woman. Of the twelve paid members four would be legal members, the same number of legal members as now existed in the case of the Commissioners. There would be four medical members, a paid chairman, and three other members. The existing paid Lunacy Commissioners, eight in number, were to be *ex-officio* members of the Board of Control. It was further proposed that an executive committee of the Board should be constituted, consisting of the chairman and not more than four paid members. All the administrative work would be executed by that committee. The existing work of the Commissioners, who will become Commissioners of the Board of Control, will continue on the same footing as at present, except that they will have added other duties under this Bill.

### REWARDS FOR CLINICAL RESEARCH.

The Moxon Gold Medal, which was founded in 1887 in memory of Dr. Walter Moxon, and which is granted every third year to the person deemed most distinguished by observation and research in clinical medicine, has been awarded to Sir David Ferrier, M.D., F.R.S. Sir David, who was knighted last year and is Emeritus Professor of Neuro-pathology at King's College, London, is an M.D. of Edinburgh, and still practises as a physician. No one nowadays can doubt the need for encouraging clinical research and observation, and the Moxon Medal, which has been established for twenty-five years, bears witness to the need then felt for honouring this branch of medicine. If that need was felt years ago it is one which is known by responsible people to-day to be increasingly real and dangerously overlooked.

### WOMEN DOCTORS AND THE WHITE SLAVE TRAFFIC.

Recent supporters of the Criminal Law Amendment Bill include women doctors, one hundred and twenty of whom are announced to have signed a memorial to Mr. Asquith in its support. These signatories may be expected to have approached the question with less of the hysteria and sentimentalism which have been aired so painfully of late, and which no doubt Sir Frederick Banbury had in mind when making some caustic criticisms a week or two ago. Far too many supporters of this measure have been carried away by the object at which it aims, and seem to have forgotten that the supposed efficacy of its provisions, which fills them with delight, may well prove to have been purchased by a disastrous interference with the liberty of the subject. For with its proposed power of arrest without a warrant the day may easily come when one or other of these same supporters may find an innocent pause at Piccadilly misinterpreted by the stern servant of the law into whose hands they propose to give such arbitrary powers. This side of the question is often forgotten: the stringency which bestows unchecked powers of arrest is a stringency of which the whole public may become the victim. Were the attendant risks of this measure clearly explained in the memorial which the women doctors were asked to sign? We expect they were ignored, and that the signatories knew of nothing and cared for nothing but an object with which they felt in sympathy. On such a subject the women members of the profession should not allow themselves rashly to be carried away.

### A SPLENDID INHALATORIUM.

By far the largest institution in Germany devoted entirely to the practical application of the latest methods of inhalation is soon to be opened at Bad Soden in the Taunus district near Frankfurt-on-the-Main. This inhalatorium is splendidly situated in the grounds of the Kurgarten of this beautiful spa, and is fitted up with the most modern appliances for conveniently carrying out inhalation treatment in its many phases. The inception and the successful development of the scheme are due to the enterprise of the local doctors, who have managed to erect and equip a magnificent building at a cost of half a million marks (£25,000). The accommodation provided will allow 250 patients to make use of every grade of inhalation therapy, from the moist variety to the driest atmosphere. There are five large rooms which will accommodate at least thirty persons each, and eighteen small rooms providing six different kinds of inhaling apparatus. The institution is under the superintendence of Dr. Henry Hughes, one of the oldest practitioners in this resort, and will decidedly increase the importance of this spa.

### THE AFFAIRS OF NORTH DEVON INFIRMARY.

Mr. C. E. Roberts Chanter, who has held the post of chairman of the house committee of the North Devon Infirmary for three years, is now retiring. At the recent annual meeting Mr. R. P. Dunn Pattison and Mr. W. J. Parkhouse referred particularly to the business aptitude with which he had faced the institution's financial difficulties on his appointment, and to the courtesy and tact with which the Friendly Society members had been treated in the person of their representative. At the same meeting a discussion took place as to the admission of contagious cases requiring operation, it being the desire of some to exclude these. Dr. J. R. Harper, Mayor of Barnstaple, took the case of diphtheria as an example, and urged that if they were going to close their doors to operations consequent upon diphtheria, a chance of saving a fair number of infantile lives would be thrown away, while the staff did not hesitate to run the risk. It was decided to postpone the discussion for twelve months. It is pointed out that an isolation hospital is badly needed at Barnstaple.

### THE AIMS OF EUGENISTS.

Major Leonard Darwin presided at the opening of the first International Eugenics Congress at the University of London last week. Many nationalities were represented, and the Congress had been organised by the Eugenics Education Society in order to make more widely known the aims of Eugenists. What are these aims? We are afraid that it must be admitted that the majority of the papers contribute nothing to their elucidation. The words "heredity" and "environment" occur with frequency, and vague allusions are made to the moral difficulties Eugenists in practice expect to meet. In the same breath it is added that all scientific witnesses of importance agree in thinking that something must be done. The Eugenists therefore are on the horns of a dilemma; if they mean business it will be by conducting certain experiments that, they continue to assert, will be regarded as immoral by an ignorant public; or, if they fear to excite lay opposition, practically no useful experiments will be open to them at all. The truth simply is that control of breeding in the human subject for any preconceived scientific purpose is held to be scandalous, regardless of the fact that the history of every dynastic family shows preconceived purposes invariably placed above personal ones. The moral, therefore, is that, until the Eugenics Education Society can teach the public that breeding, in the widest sense, for preconceived purposes is not objectionable, but already approved by the most respectable precedents, it will achieve nothing. Here is a task that Major Darwin would do well to attempt. Even Havelock Ellis seems to be confused. In his new book, "The Task of Social Hygiene," he quotes approvingly the remark, "We want men like William James and Francis Galton," while adding on the next page, "We may therefore quite put aside all discussion of Eugenics as a sort of higher cattle breeding." Yet the practical proposals which follow, medical certificates, eugenic pedigrees, and so forth, that rightly correspond to stock records, contradict this view. It is necessary to insist on clear thinking, for Parliament contains so few members who are capable of discussing these problems in a sane manner that it is likely that Bills which deal with vital questions may be passed in a moment of enthusiasm, to leave the future generation with a weight of legislation whose tendency is reactionary in the extreme.

### A DEPARTURE IN HOSPITAL ADVERTISING.

The following curious advertisement appeared in a London newspaper on Tuesday morning: "A London hospital, of ancient foundation, bestowing inestimable benefits upon 12,000 persons each year, has suffered such neglect at the hands of the charitable public for a long course of years that it is in grievous difficulties, from which, however, a comparatively small sum would free it. To the generous benefactor who would save it would be extended the privilege, if desired, of renaming the hospital (*e.g.*, in memory of a departed relative or friend). Full particulars can be obtained from the Honorary Secretary (care of a firm of solicitors), 35 Queen Victoria Street, E.C." Advertisements for the naming of beds, cots, or wards are, of course, common, but it is not often that recourse is had to a general invitation to rename a whole institution. It would be very interesting to know what is the number and nature of replies received. In these days of mushroom fortunes there should be many anxious to take advantage of this offer, and the smaller charitable institutions in difficulties will no doubt watch carefully any institutional rechristening to judge of the success of this attempt.

### AN EXCHEQUER GRANT FOR COUNTY COUNCIL SANATORIA.

A deputation representing the County Councils Association waited on Mr. Lloyd George and Mr. Burns on Tuesday to find out on what assistance the county councils may rely from the Exchequer to carry out the schemes for dealing with tuberculosis under the Insurance Act. Sir W. Ryland Adkins, M.P., said that the county councils were anxious to put sanatorium benefit into force, but whether they could do so must depend upon the Treasury, the assistance of which they hoped would go direct to the local authorities from the Local Government Board, and not through the Insurance Committees. Mr. Henry Hobhouse suggested that the Exchequer should bear 75 per cent. of the annual cost of maintaining the institutions to be provided. In reply, Mr. Lloyd George said that county councils were entitled to an answer before they began to spend money. The amount he promised to state later (unfortunately, after we have gone to press). The method of payment, he said, must very largely be a matter of arrangement with the Insurance Committees.

### FORTY YEARS OF PUBLIC HEALTH.

The position of Medical Officer of Health for the City of Newcastle-on-Tyne has been rendered vacant by the retirement of Dr. Henry Edward Armstrong, who has held the position for thirty-nine years. Dr. Armstrong, who took his M.R.C.S. in 1864, has been examiner and lecturer on public health to the University of Durham College of Medicine, and it was Durham University which conferred on him the degree of Doctor of Hygiene, *honoris causa*, in 1891. Formerly he was medical superintendent of the Newcastle City Infectious Diseases and Smallpox Hospital. Among his numerous reports and publications we may note his "Sketch of the Sanitary History" of Newcastle, and his "Education, Training, and Qualification of Medical Officers of Health." Since his appointment in 1873 public health work has developed enormously in direction, extent, and importance, and the varied scope of the work seems to attract good men, to whom it offers responsibilities and security not easily paralleled in private practice.

### MR. BEIT'S GIFT TO A CAMBRIDGE HOSPITAL.

The Treasurers of the new Research Hospital at Cambridge, of which we published an illustrated account a few weeks ago, have received £500 from Mr. Otto Beit towards the sum of £8,000 which they hope to raise for the endowment of the hospital. They announce further that an anonymous medical man has offered to give a scholarship of the value of £150 a year for three years for research on rheumatoid arthritis at the Research Hospital. Gifts are luckily infectious, often proving to be the best kind of advertisement, and we therefore hope that Mr. Otto Beit's gift may become widely known, so that this original institution may shortly receive the endowment which is necessary for its work.

### THIS WEEK'S DRUG MARKET.

The probability of an advance in the price of quinine is being freely talked about; it is rumoured that an arrangement is pending between the German and English manufacturers of the alkaloid on the one hand, and the producers of cinchona bark in Java on the other, under which the price of the bark would be regulated. Reports of a similar nature have so often proved to be wrong that it is difficult to know whether to give credence to the present report or not. In any case, the present price of quinine is sufficiently low to tempt those who think the agreement may be arrived at to purchase a fair stock of the drug; there seems to be no likelihood of a reduction in price in the near future. The termination of the labour troubles at the docks is naturally welcomed by drug importers and merchants, but as this is the quietest season of the year it is not likely that the cessation of the strike will have an immediately noticeable effect on the trade. There have been few important changes in price during the week. Citronella oil is dearer. Cream of tartar is tending upwards in value. The position of opium is unchanged, buyers still expecting that much lower prices will soon be quoted. Orris root is dearer. At the recent public sale of drugs in Mincing Lane, Japanese refined camphor sold at rather lower prices, croton seed sold at dear rates, and ipecacuanha was rather dearer.

